# Evaluation of circulating miRNAs during late pregnancy in the mare

**DOI:** 10.1371/journal.pone.0175045

**Published:** 2017-04-07

**Authors:** Shavahn C. Loux, Kirsten E. Scoggin, Jason E. Bruemmer, Igor F. Canisso, Mats H. T. Troedsson, Edward L. Squires, Barry A. Ball

**Affiliations:** 1 Department of Veterinary Science, University of Kentucky, Lexington, KY, United States of America; 2 Department of Animal Sciences, Colorado State University, Fort Collins, CO, United States of America; 3 Department of Veterinary Clinical Medicine, College of Veterinary Medicine, University of Illinois, Urbana, IL, United States of America; University of Florida, UNITED STATES

## Abstract

MicroRNAs (miRNAs) are small, non-coding RNAs which are produced throughout the body. Individual tissues tend to have a specific expression profile and excrete many of these miRNAs into circulation. These circulating miRNAs may be diagnostically valuable biomarkers for assessing the presence of disease while minimizing invasive testing. In women, numerous circulating miRNAs have been identified which change significantly during pregnancy-related complications (e.g. chorioamnionitis, eclampsia, recurrent pregnancy loss); however, no prior work has been done in this area in the horse. To identify pregnancy-specific miRNAs, we collected serial whole blood samples in pregnant mares at 8, 9, 10 m of gestation and post-partum, as well as from non-pregnant (diestrous) mares. In total, we evaluated a panel of 178 miRNAs using qPCR, eventually identifying five miRNAs of interest. One miRNA (miR-374b) was differentially regulated through late gestation and four miRNAs (miR-454, miR-133b, miR-486-5p and miR-204b) were differentially regulated between the pregnant and non-pregnant samples. We were able to identify putative targets for the differentially regulated miRNAs using two separate target prediction programs, miRDB and Ingenuity Pathway Analysis. The targets for the miRNAs differentially regulated during pregnancy were predicted to be involved in signaling pathways such as the STAT3 pathway and PI3/AKT signaling pathway, as well as more endocrine-based pathways, including the GnRH, prolactin and insulin signaling pathways. In summary, this study provides novel information about the changes occurring in circulating miRNAs during normal pregnancy, as well as attempting to predict the biological effects induced by these miRNAs.

## Introduction

MicroRNAs (miRNAs) are small, non-coding RNAs whose expression varies by tissue, with individual tissues exhibiting unique miRNA fingerprints [1]. The primary biological function of miRNAs is to repress translation of messenger RNAs (mRNA) by binding to highly complementary targets, typically on the 3’ untranslated region. Degradation of the target mRNA may or may not occur, depending on the degree of complementarity [1]. Many of these miRNAs are released into circulation, where they are bound to carrier proteins or lipoproteins such as high-density lipoprotein, or, more commonly, contained within membrane-bound vesicles known as exosomes [2, 3]. Their presence and longevity in circulation has made them attractive targets as biomarkers for a variety of conditions, including pregnancy disorders [4].

The recent interest surrounding miRNAs as biomarkers is well founded for a number of reasons. First, they are exceptionally stable within circulation with an estimated half-life of more than five days [5]. Additionally, their expression changes significantly throughout the course of many diseases and physiological conditions, including cancer [3, 6], cardiovascular disease [7], metabolic diseases (e.g. diabetes) [8], skeletal diseases [9], and pregnancy disorders [10]. Pregnancy-associated diseases are of particular interest due to the difficulties in diagnosing many of these diseases in the early stages combined with their potentially catastrophic consequences in any species. Earlier diagnosis allows for earlier intervention and a more favorable outcome.

In humans, circulating miRNA levels have been shown to change significantly during preeclampsia, ectopic pregnancy, intra-uterine growth restriction and pre-term labor [11, 12]. There has been no information about the expression of circulating miRNAs in late-term equine pregnancy; however, a recent study identified twelve exosomal circulating miRNAs proposed to be involved with maternal recognition of pregnancy [13]. Circulating miRNAs have tremendous diagnostic potential for many similar pregnancy-related complications affecting horses and humans, including fetal growth restriction and placental infection. Understanding the physiological changes in miRNA expression during normal gestation gives us important information about the role of miRNAs during pregnancy.

To the best of our knowledge, there has been no previous research on the normal expression of circulating miRNAs in whole blood from mares during late gestation. Therefore, we have evaluated miRNA expression profiles throughout late gestation and compared them to non-pregnant mares, as well as evaluating physiological changes through late gestation.

## Materials and methods

All animal procedures were prospectively approved by and completed in accordance with the Institutional Animal Care and Use Committee of the University of Kentucky (Protocol# 2010–0769). All horses used in this study were light-breed mares owned by the University of Kentucky, aquired through the breeding program or by donation. Mares ranged from 350–550 kg and were between 7 and 16 years of age. Mares were housed on pasture with free-choice grass hay available at all times. All horses were retained following the completion of this study. Gestational age was determined using the day of ovulation (Day 0).

### Experimental design

Whole blood was collected using PAXgene blood collection tubes (PreAnalytiX; Hombrechtikon, Switzerland) serially from mares at 8, 9, 10 m gestation & post-partum (n = 5) as well as in mares at 9 d diestrus (n = 4) to compare circulating miRNAs between pregnant and non-pregnant mares, as well as to determine stability of miRNA expression through late gestation. Post-partum samples were taken 14–31 days post-foaling. PAXgene tubes were immediately frozen at -20°C. Real-time PCR was used to evaluate the relative expression of 178 mature equine miRNAs within these samples (S1 Table). All mares were confirmed to have normal pregnancies by ultrasonography at the sampling times and thorough pathologic placental evaluation postpartum.

### RNA purification/qPCR

RNA was purified using the PAXgene blood miRNA kit (Qiagen, Germantown, MD, USA) per manufacturer’s instructions, then stored at -20°C until use. RNA purity and concentration were assessed using the NanoDrop 2000 spectrophotometer (ThermoScientific, Waltham, MA, USA), with 260/280 ratios above 1.8 and 260/230 ratios above 2.0 being considered acceptable.

Libraries of cDNA were created using the miScript Reverse Transcription kit (Qiagen) with the HiSpec® buffer to select for mature miRNAs. A total of 400 ng RNA was used per reaction, following manufacturer’s instructions. In short, 4 μL 5x HiSpec buffer, 2 μL 10x miScript Nucleics Mix, 2 μL miScript Reverse Transcriptase Mix were used, with nuclease-free water added to bring the final volume to 20 μL. An additional aliquot was made with water in place of the RNA for a negative control. Tubes were held at 37°C for 60 min followed by 5 min at 95°C, then cooled to 4°C where it was held until use.

Reactions were immediately prepared for qPCR using the miScript SYBR Green PCR kit (Qiagen). In short, each reaction consisted of 3 μL 2x QuantiTect SYBR Green PCR Master Mix, 0.6 μL 10x miScript Universal Primer, 1.34 μL nuclease-free H_2_O, 1.5 μL miRNA-specific forward primer and 0.06 μL cDNA. All reactions were performed in duplicate. The RNA-free reverse transcription reaction and a cDNA-free PCR reaction were used as negative controls. Thermal cycling took place on a LightCycler 480 PCR system (Roche, Indianapolis, IN, USA), and consisted of an initial incubation at 95°C for 15 min followed by 40 cycles at 95°C for 15 sec, 55°C for 30 sec and 72°C for 30 sec. Melt curve analysis was performed to confirm amplification of a single cDNA product.

Data were normalized by ΔCt = Ct_(target)_−Ct_(mean of sample)_. This method of normalization has been shown to be superior to identifying select housekeeping genes for large-scale miRNA expression studies [14].

### MicroRNA target evaluation

Predicted targets of differentially expressed miRNAs were assessed using miRDB [15], with mRNAs with a target prediction score (TPS) of ≥ 80 selected for further analysis as suggested by the manufacturer. The predicted mRNA targets for each miRNA are listed in S2 File.

To assess which pathways were affected by the altered miRNA expression, predicted target mRNAs were analyzed using Ingenuity Pathway Analysis (Qiagen, Redwood City, CA, USA). The mRNAs were grouped based on the status of the targeting miRNA, and a core analysis was run on each group. Canonical pathways were derived from each core analysis.

### Statistics

Data analyses were performed using JMP 11 (SAS Institute, Cary, NC, USA). Variability of miRNA expression through late gestation was analyzed using one-way ANOVA adjusted for false-discovery rate with the Benjamini-Hochberg adjustment (P < 0.1), with the ΔCt value as the Y variable and the stage of gestation, or pregnancy status as the X variable [16]. Post-hoc analysis was performed using student’s t-test for pregnant vs diestrus (P < 0.05).

## Results

### Late gestation/diestrus analysis

**Fig 1:**
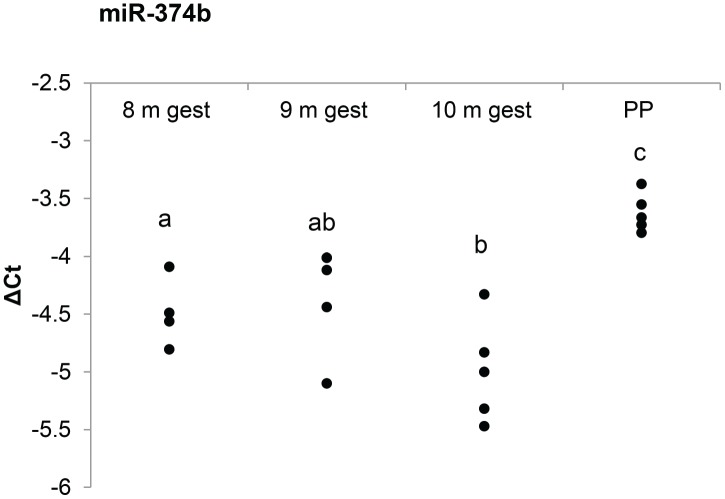
Differential expression of miR-374b through late gestation. Expression of circulating miRNAs was analyzed using quantitative PCR through late gestation (8, 9 or 10 months) and post-partum (PP). One miRNA, miR-374b, was found to be differentially regulated. Data shown represent the ΔCt value (dCt), calculated as ΔCt = Ct_(target)_−Ct_(mean of sample)_, with each dot representative of a separate sample. A lower ΔCt value indicates a higher expression level; for each 1-point reduction in ΔCt, an approximately 2-fold increase in expression can be assumed. Significantly different samples are indicated by varying superscripts.

Only a single miRNA derived from whole blood was differentially regulated through the late stages of gestation, miR-374b. Post-hoc analysis confirmed expression of miR-374b was significantly increased at all stages (8, 9 and 10 m) of gestation when compared to post-partum (P<0.05; Fig 1). Additionally, the expression level of miR-374b at 10 m gestation was upregulated compared to 8 m gestation. When miR-374b was analyzed with mirDB, 247 putatively targeted mRNAs with a target prediction score of ≥ 80 were identified (S2 File). These mRNAs are hypothesized to be involved in 261 canonical pathways; the top twenty pathways being presented in Table 1, with a full list of canonical pathways found in S3 File. The top pathways included axonal guidance signaling, TGF-β signaling, insulin receptor signaling, regulation of the epithelial-mesenchymal transition pathway, relaxin signaling and estrogen receptor signaling.

**Table 1:** Top twenty pathways predicted for the miRNA differentially regulated through late gestation (miR-374b).

Ingenuity Canonical Pathway	-log (p-value)
VDR/RXR Activation	4.28
tRNA Splicing	2.93
Axonal Guidance Signaling	2.39
TGF-β Signaling	2.31
Insulin Receptor Signaling	2.1
GABA Receptor Signaling	2.01
Regulation of the Epithelial-Mesenchymal Transition Pathway	2.01
ErbB2-ErbB3 Signaling	1.97
Relaxin Signaling	1.96
Paxillin Signaling	1.89
Transcriptional Regulatory Network in Embryonic Stem Cells	1.86
Neurotrophin/TRK Signaling	1.81
PTEN Signaling	1.76
Protein Kinase A Signaling	1.68
G-Protein Coupled Receptor Signaling	1.68
Prolactin Signaling	1.68
Valine Degradation I	1.67
Estrogen Receptor Signaling	1.63
Neuregulin Signaling	1.63
GADD45 Signaling	1.63

**Fig 2:**
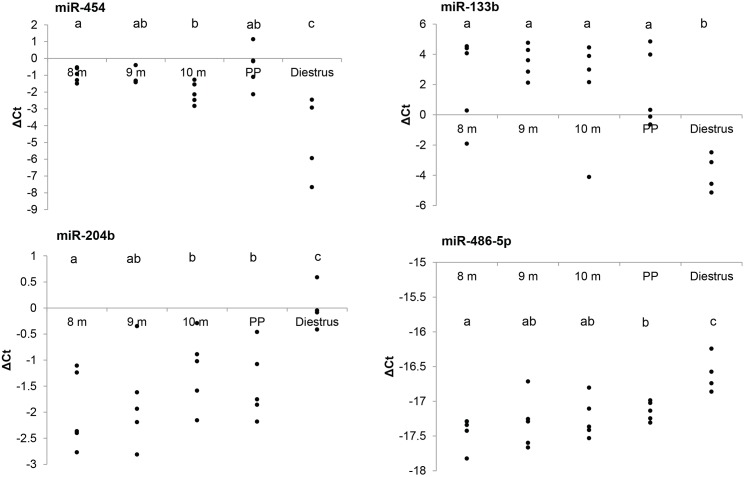
Differential expression of circulating miRNAs in pregnant mares. Expression of circulating miRNAs was analyzed using quantitative PCR to identify miRNAs with significantly different expression during pregnancy. Samples were grouped into pregnant (8, 9, 10 m gestation, postpartum (PP)) or non-pregnant (diestrus) samples and analyzed by one-way ANOVA corrected for false discovery rate (Benjamini-Hochberg; P < 0.05), with post-hoc analysis performed by student’s t-test (P < 0.1). Data were normalized by ΔCt = Ct_(target)_−Ct_(mean of the sample)_, with each point representing the ΔCt for an individual mare. A lower ΔCt value indicates a higher expression level; for each 1-point reduction in ΔCt, an approximately 2-fold increase in expression can be assumed. Significantly different samples are indicated by varying superscripts.

We identified four miRNAs which had altered expression between pregnant and non-pregnant mares, including two miRNAs which were up-regulated during pregnancy (miR-204b, miR-486-5p) and two which were down-regulated (miR-454, miR-133b; Fig 2). Post-hoc analysis revealed that each stage of gestation (8 m, 9 m, 10 m and post-partum) was significantly different than diestrus for each of these miRNAs.

The number of predicted target mRNAs for each miRNA ranged from 59 (miR-486-5p) to 271 (miR-454; S2 File). Twenty-nine mRNAs were the predicted targets of more than a single miRNA. Intriguingly, 23 / 29 of these shared mRNA targets were targeted by both an up-regulated miRNA and a down-regulated miRNA, although this may be simply due to the relative number of predicted targets for each miRNA.

The mRNA targets of miRNAs differentially regulated in pregnancy were analyzed to determine their putative role in canonical pathways by Ingenuity Pathway Analysis, with a total of 332 pathways predicted (S3 File). The top twenty pathways are presented in Table 2. These pathways are primarily signaling pathways, with 17 of the top 20 pathways representing signaling pathways, including the STAT3 pathway, prolactin signaling, GnRH signaling, PI3/AKT signaling and JAK/stat signaling. Four of the pathways were present in the top 20 pathways for both miRNAs changing through late gestation, as well as for miRNAs changing between pregnant and non-pregnant samples. These duplicated pathways included the epidermal growth factor receptor family (ErbB2-ErbB3 signaling), the insulin receptor signaling pathway, the prolactin signaling pathway and the phosphatase and tensin homolog (PTEN) signaling pathway.

**Table 2:** Top twenty pathways predicted for miRNAs differentially regulated during pregnancy (miR-133b, miR-204b, miR-454, miR-486-5p).

Ingenuity Canonical Pathways–Pregnant Mares	-log (p-value)
STAT3 Pathway	5.35
PPARα/RXRα Activation	4.99
Prolactin Signaling	4.76
PTEN Signaling	4.41
IGF-1 Signaling	4.32
Glioblastoma Multiforme Signaling	4.14
JAK/Stat Signaling	4.05
CTLA4 Signaling in Cytotoxic T Lymphocytes	4.03
PAK Signaling	3.94
Glioma Signaling	3.59
GNRH Signaling	3.52
ErbB2-ErbB3 Signaling	3.41
EGF Signaling	3.41
Renin-Angiotensin Signaling	3.24
Insulin Receptor Signaling	3.2
Mouse Embryonic Stem Cell Pluripotency	3.15
PI3K/AKT Signaling	3.14
GDNF Family Ligand-Receptor Interactions	3.05
Telomerase Signaling	3.05
Non-Small Cell Lung Cancer Signaling	3.01

## Discussion

We have identified, for the first time, pregnancy-associated circulating miRNAs in the late–pregnant mare. Due to their presence in peripheral circulation, these miRNAs are easily assessed with a simple blood test. Although their diagnostic ability is still being evaluated, they have potential to be used as biomarkers for the assessment of gestational well-being.

With any study on miRNAs, it is important to note the type of sample used, as well as the methodology employed for extraction and evaluation. This is particularly true for blood, as results vary significantly depending on whether plasma, serum, whole blood or exosomes are being targeted [17]. We chose to collect whole blood samples in PAXgene tubes because there is evidence that storing blood in RNA specific storage kits such as PAXgene tubes maintains the integrity of the RNA more effectively than serum stored at -80°C [18], as well as simplifying collection procedures. That said, there are downfalls of analyzing the miRNA composition of whole blood as compared to serum, plasma or exosomes. Whole blood contains not only miRNAs from the target tissues, but also miRNAs from each individual cellular components of blood including erythrocytes [19], leukocytes [20] and platelets [21]. The miRNAs derived from the cellular components of blood provide background noise, making it more difficult to identify changes in placental-derived miRNAs. Moreover, without separating leukocyte-derived miRNAs from placental-derived miRNAs, we may be inadvertently biasing our samples towards inflammatory miRNAs rather than placenta-specific targets.

Of the four miRNAs differentially regulated during pregnancy, two were down-regulated. This is a significantly larger proportion than previously reported in human literature which has primarily focused on miRNAs with increasing expression during gestation [22–25]. This discrepency may be due to study design, as one human study identified highly abundant miRNAs from the placenta, then looked for their presence in circulation [22], while another only reported up-regulated miRNAs [25] in a search for pregnancy-specific miRNAs. This difference in methodology is interesting in part due to the differences in expression we noted between our up- and down-regulated miRNAs. Within these four miRNAs, much larger changes in expression were seen in the down-regulated miRNAs. Looking at miR-133b, large fold-changes between pregnancy and diestrus were consistently noted, ranging from a 45 X decrease during the post-partum period to a 164 X decrease at 9 m gestation. The other down-regulated miRNA (miR-454) was 12.9 X lower on average (range of 6.5–19.2 X). The two up-regulated miRNAs (miR-204b and miR-486-5p) had an average increase of only 2.4 X (range– 1.44–3.96 X; Fig 2). This is not surprising biologically, as miRNAs are responsible for the down-regulation of mRNA, and mRNA expression increases overall during pregnancy. Although it is difficult to make broad conclusions based on only four differentially regulated miRNAs, it is an intriguing trend.

The two miRNAs we identified as down-regulated during late gestation have not previously been identified as being important during pregnancy, although this could be largely due to prior study design in humans. Out of our up-regulated miRNAs, miR-486-5p has not been previously reported to be pregnancy-related, despite being the most highly expressed miRNA within our samples. Overall, expression levels of miR-486-5p increased by an average of 1.6-fold during pregnancy. Our other up-regulated miRNA, miR-204b, has an identical mature sequence to hsa-miR-204, which has been shown to be up-regulated in pre-eclampsia [26], potentially due to its role in suppressing trophoblast-like cell invasion [27].

Although our down-regulated miRNAs, miR-133b and miR-454, are certainly not pregnancy specific, their down-regulation during pregnancy allows for an increase in the translation of numerous mRNAs, including X-linked RNA binding motif protein (RBMX) which is implicated in regulation of gene transcription and alternative splicing, as well as repairing double-stranded DNA breaks [28, 29] and DEAD (Asp-Glu-Ala-Asp) box helicase 6 (DDX6) which maintains adult progenitor cell function [30].

Together, the mRNAs targeted by the pregnancy-related miRNAs are predicted to be a part of 332 canonical pathways by Ingenuity Pathway Analysis (S3 File). Included in the top twenty predicted pathways are the STAT3 pathway, JAK/stat signaling, prolactin signaling, PI3K/AKT signaling, GnRH signaling, and mouse embryonic stem cell pluripotency markers. The purported effect of these miRNAs on the prolactin and GnRH signaling pathways is interesting due to the essential role of these pathways in pregnancy.

Although GnRH hasn’t been studied extensively in late pregnancy, its ability to stimulate gonadotropins and with that, steroid hormones such as estrogens and progestogens, is well established [31]. Prolactin is perhaps best known for its role in lactation [32]; however, it is also responsible for activating several other pathways such as the JAK-STAT pathway, including STAT3 [33, 34]. The function of STAT3 is well established in pregnancy, including involvement with embryonic implantation [35, 36], angiogenesis [37], and parturition [38, 39].

PI3K/AKT signaling has been shown to play a role in trophectoderm migration [40] and placental resource allocation [41], as well as being implicated in embryonic neurogenesis and angiogenesis [40, 42]. Many of these pathways also work in conjunction with one another; the PI3K and prolactin signaling pathways have been shown to increase pancreatic islet mass and sensitivity to glucose during pregnancy [43]; likely influencing insulin receptor signaling, another affected pathway, simultaneously.

Although we have some information about the likely function of these differentially expressed miRNAs, we do need to proceed with caution. Many of our miRNAs did not exist in the Ingenuity Pathway Analysis database, so to get an equivocal analysis we derived predicted mRNA targets using miRDB, a human dataset [15]. Since mRNAs are not identical across species, it is possible that some of these mRNAs are not present in the horse or that they do not contain the miRNA target sequences. However, miRNA annotation is highly conserved and identical miRNAs are assigned the same number regardless of organism, increasing the likelihood of cross-species applicability [44]. Additionally, we only selected mRNA targets with a predicted target score of 80 or above, a level which the designers considered “very probable” to be a valid target [15]. Additional caution should be exercised with the designation of pathways by Ingenuity Pathway Analysis. Many of the designated pathways contain overlapping molecules, so it is possible for multiple pathways to be considered influenced even though it is unlikely for many of those same pathways to be present or applicable to the work. For example, it is unlikely that our miRNAs are affecting glioblastoma multiform signaling, despite this being the top predicted pathway for pregnancy-related miRNAs. Despite these concerns, most of the predicted pathways are not unexpected and provide valuable information about the biology of these differentially regulated miRNAs.

In conclusion, we have identified eight circulating miRNAs which changed significantly during gestation. The pregnancy-specific miRNAs are predicted to target mRNAs involved in signaling pathways, particularly those which are involved in placentation, angiogenesis and endocrinology of pregnancy. This study not only identifies potential biomarkers for placental function in late gestation, it also provides information about their putative targets and what pathways may be affected during these periods.

## Supporting information

S1 File Raw and normalized Ct values for qPCR. (XLSX)

S2 File Putative mRNA targets. Putative mRNA targets for individual miRNAs as predicted by miRDB [15]. Only mRNAs with a predicted target score of ≥80 are included. Includes tab for shared mRNAs which were targeted by multiple miRNAs.
(XLSX)

S3 File Predicted canonical pathways. Ingenuity Pathway Analysis (IPA) was used to predict canonical pathways affected by putative mRNA targets of differentially regulated miRNAs. Putative mRNA targets were identified using miRDB [15], and mRNAs with a predicted target score of ≥80 were pooled and analyzed via IPA core analysis.
(XLS)

S1 Table Primer sequences. Sequences of 178 primers used for qPCR analysis.
(DOCX)

## References

[1] Wilczynska A, Bushell M. The complexity of miRNA-mediated repression. Cell death and differentiation. 2015;22(1):22–33. Epub 2014/09/06. PubMed Central PMCID: PMCPMC4262769. doi: 10.1038/cdd.2014.112 25190144 10.1038/cdd.2014.112 PMC4262769

[2] Ghai V, Wang K. Recent progress toward the use of circulating microRNAs as clinical biomarkers. Archives of toxicology. 2016. Epub 2016/09/03. 10.1007/s00204-016-1828-2 27585665

[3] Singh R, Ramasubramanian B, Kanji S, Chakraborty AR, Haque SJ, Chakravarti A. Circulating microRNAs in cancer: Hope or hype? Cancer letters. 2016;381(1):113–21. Epub 2016/07/30. doi: 10.1016/j.canlet.2016.07.002 27471105 10.1016/j.canlet.2016.07.002

[4] Hromadnikova I, Kotlabova K, Hympanova L, Krofta L. Gestational hypertension, preeclampsia and intrauterine growth restriction induce dysregulation of cardiovascular and cerebrovascular disease associated microRNAs in maternal whole peripheral blood. Thrombosis research. 2016;137:126–40. Epub 2015/12/04. doi: 10.1016/j.thromres.2015.11.032 26632513 10.1016/j.thromres.2015.11.032

[5] Gantier MP, McCoy CE, Rusinova I, Saulep D, Wang D, Xu D, et al Analysis of microRNA turnover in mammalian cells following Dicer1 ablation. Nucleic acids research. 2011;39(13):5692–703. Epub 2011/03/31. PubMed Central PMCID: PMCPmc3141258. doi: 10.1093/nar/gkr148 21447562 10.1093/nar/gkr148 PMC3141258

[6] He Y, Lin J, Kong D, Huang M, Xu C, Kim TK, et al Current State of Circulating MicroRNAs as Cancer Biomarkers. Clinical chemistry. 2015;61(9):1138–55. Epub 2015/09/01. doi: 10.1373/clinchem.2015.241190 26319452 10.1373/clinchem.2015.241190

[7] Maegdefessel L. The emerging role of microRNAs in cardiovascular disease. Journal of internal medicine. 2014;276(6):633–44. Epub 2014/08/28. doi: 10.1111/joim.12298 25160930 10.1111/joim.12298

[8] Sethupathy P. The Promise and Challenge of Therapeutic MicroRNA Silencing in Diabetes and Metabolic Diseases. Current diabetes reports. 2016;16(6):52 Epub 2016/04/27. PubMed Central PMCID: PMCPMC4844637. doi: 10.1007/s11892-016-0745-3 27112956 10.1007/s11892-016-0745-3 PMC4844637

[9] Seeliger C, Balmayor ER, van Griensven M. miRNAs Related to Skeletal Diseases. Stem cells and development. 2016;25(17):1261–81. Epub 2016/07/16. doi: 10.1089/scd.2016.0133 27418331 10.1089/scd.2016.0133

[10] Mouillet JF, Ouyang Y, Coyne CB, Sadovsky Y. MicroRNAs in placental health and disease. American journal of obstetrics and gynecology. 2015;213(4 Suppl):S163–72. Epub 2015/10/03. PubMed Central PMCID: PMCPMC4592520. doi: 10.1016/j.ajog.2015.05.057 26428496 10.1016/j.ajog.2015.05.057 PMC4592520

[11] Zhao Z, Moley KH, Gronowski AM. Diagnostic potential for miRNAs as biomarkers for pregnancy-specific diseases. Clinical biochemistry. 2013;46(10–11):953–60. Epub 2013/02/12. doi: 10.1016/j.clinbiochem.2013.01.026 23396163 10.1016/j.clinbiochem.2013.01.026

[12] Cretoiu D, Xu J, Xiao J, Suciu N, Cretoiu SM. Circulating MicroRNAs as Potential Molecular Biomarkers in Pathophysiological Evolution of Pregnancy. Disease markers. 2016;2016:3851054 Epub 2016/08/06. PubMed Central PMCID: PMCPMC4967453. doi: 10.1155/2016/3851054 27493447 10.1155/2016/3851054 PMC4967453

[13] Klohonatz KM, Cameron AD, Hergenreder JR, da Silveira JC, Belk AD, Veeramachaneni DN, et al Circulating miRNAs as Potential Alternative Cell Signaling Associated with Maternal Recognition of Pregnancy in the Mare. Biology of reproduction. 2016. Epub 2016/10/21. 10.1095/biolreprod.116.142935 27760749

[14] Mestdagh P, Van Vlierberghe P, De Weer A, Muth D, Westermann F, Speleman F, et al A novel and universal method for microRNA RT-qPCR data normalization. Genome biology. 2009;10(6):R64 Epub 2009/06/18. PubMed Central PMCID: PMCPMC2718498. doi: 10.1186/gb-2009-10-6-r64 19531210 10.1186/gb-2009-10-6-r64 PMC2718498

[15] Wong N, Wang X. miRDB: an online resource for microRNA target prediction and functional annotations. Nucleic acids research. 2015;43(Database issue):D146–52. Epub 2014/11/08. PubMed Central PMCID: PMCPMC4383922. doi: 10.1093/nar/gku1104 25378301 10.1093/nar/gku1104 PMC4383922

[16] Benjamini Y, Hochberg Y. Controlling the False Discovery Rate: A Practical and Powerful Approach to Multiple Testing. Journal of the Royal Statistical Society Series B (Methodological). 1995;57(1):289–300.

[17] Backes C, Meese E, Keller A. Specific miRNA Disease Biomarkers in Blood, Serum and Plasma: Challenges and Prospects. Molecular diagnosis & therapy. 2016. Epub 2016/07/06. 10.1007/s40291-016-0221-4 27378479

[18] Unger L, Fouche N, Leeb T, Gerber V, Pacholewska A. Optimized methods for extracting circulating small RNAs from long-term stored equine samples. Acta veterinaria Scandinavica. 2016;58(1):44 Epub 2016/07/01. PubMed Central PMCID: PMCPMC4928274. doi: 10.1186/s13028-016-0224-5 27356979 10.1186/s13028-016-0224-5 PMC4928274

[19] Doss JF, Corcoran DL, Jima DD, Telen MJ, Dave SS, Chi JT. A comprehensive joint analysis of the long and short RNA transcriptomes of human erythrocytes. BMC genomics. 2015;16:952 Epub 2015/11/18. PubMed Central PMCID: PMCPMC4647483. doi: 10.1186/s12864-015-2156-2 26573221 10.1186/s12864-015-2156-2 PMC4647483

[20] Brogaard L, Heegaard PM, Larsen LE, Mortensen S, Schlegel M, Durrwald R, et al Late regulation of immune genes and microRNAs in circulating leukocytes in a pig model of influenza A (H1N2) infection. Scientific reports. 2016;6:21812 Epub 2016/02/20. PubMed Central PMCID: PMCPMC4759598. doi: 10.1038/srep21812 26893019 10.1038/srep21812 PMC4759598

[21] Pontes TB, Moreira-Nunes Cde F, Maues JH, Lamarao LM, de Lemos JA, Montenegro RC, et al The miRNA Profile of Platelets Stored in a Blood Bank and Its Relation to Cellular Damage from Storage. PloS one. 2015;10(6):e0129399 Epub 2015/06/30. PubMed Central PMCID: PMCPMC4486185. doi: 10.1371/journal.pone.0129399 26121269 10.1371/journal.pone.0129399 PMC4486185

[22] Chim SS, Shing TK, Hung EC, Leung TY, Lau TK, Chiu RW, et al Detection and characterization of placental microRNAs in maternal plasma. Clinical chemistry. 2008;54(3):482–90. Epub 2008/01/26. doi: 10.1373/clinchem.2007.097972 18218722 10.1373/clinchem.2007.097972

[23] Ladomery MR, Maddocks DG, Wilson ID. MicroRNAs: their discovery, biogenesis, function and potential use as biomarkers in non-invasive prenatal diagnostics. Int J Mol Epidemiol Genet. 2011;2(3):253–60. Epub 2011/09/15. PubMed Central PMCID: PMCPmc3166153. 21915364 PMC3166153

[24] Miura K, Miura S, Yamasaki K, Higashijima A, Kinoshita A, Yoshiura K, et al Identification of pregnancy-associated microRNAs in maternal plasma. Clinical chemistry. 2010;56(11):1767–71. Epub 2010/08/24. doi: 10.1373/clinchem.2010.147660 20729298 10.1373/clinchem.2010.147660

[25] Gilad S, Meiri E, Yogev Y, Benjamin S, Lebanony D, Yerushalmi N, et al Serum microRNAs are promising novel biomarkers. PloS one. 2008;3(9):e3148 Epub 2008/09/06. PubMed Central PMCID: PMCPMC2519789. doi: 10.1371/journal.pone.0003148 18773077 10.1371/journal.pone.0003148 PMC2519789

[26] Choi SY, Yun J, Lee OJ, Han HS, Yeo MK, Lee MA, et al MicroRNA expression profiles in placenta with severe preeclampsia using a PNA-based microarray. Placenta. 2013;34(9):799–804. Epub 2013/07/09. doi: 10.1016/j.placenta.2013.06.006 23830491 10.1016/j.placenta.2013.06.006

[27] Yu Y, Wang L, Liu T, Guan H. MicroRNA-204 suppresses trophoblast-like cell invasion by targeting matrix metalloproteinase-9. Biochemical and biophysical research communications. 2015;463(3):285–91. Epub 2015/05/25. doi: 10.1016/j.bbrc.2015.05.052 26003727 10.1016/j.bbrc.2015.05.052

[28] Adamson B, Smogorzewska A, Sigoillot FD, King RW, Elledge SJ. A genome-wide homologous recombination screen identifies the RNA-binding protein RBMX as a component of the DNA-damage response. Nature cell biology. 2012;14(3):318–28. Epub 2012/02/22. PubMed Central PMCID: PMCPMC3290715. doi: 10.1038/ncb2426 22344029 10.1038/ncb2426 PMC3290715

[29] Kanhoush R, Beenders B, Perrin C, Moreau J, Bellini M, Penrad-Mobayed M. Novel domains in the hnRNP G/RBMX protein with distinct roles in RNA binding and targeting nascent transcripts. Nucleus (Austin, Tex). 2010;1(1):109–22. Epub 2011/02/18. PubMed Central PMCID: PMCPMC3035123. 10.4161/nucl.1.1.10857 PMC3035123 21327109

[30] Wang Y, Arribas-Layton M, Chen Y, Lykke-Andersen J, Sen GL. DDX6 Orchestrates Mammalian Progenitor Function through the mRNA Degradation and Translation Pathways. Molecular cell. 2015;60(1):118–30. Epub 2015/09/29. PubMed Central PMCID: PMCPMC4592480. doi: 10.1016/j.molcel.2015.08.014 26412305 10.1016/j.molcel.2015.08.014 PMC4592480

[31] Rudolph LM, Bentley GE, Calandra RS, Paredes AH, Tesone M, Wu TJ, et al Peripheral and Central Mechanisms Involved in the Hormonal Control of Male and Female Reproduction. Journal of neuroendocrinology. 2016;28(7). Epub 2016/06/23. 10.1111/jne.12405 PMC5146987 27329133

[32] Arendt LM, Kuperwasser C. Form and function: how estrogen and progesterone regulate the mammary epithelial hierarchy. Journal of mammary gland biology and neoplasia. 2015;20(1–2):9–25. Epub 2015/07/21. PubMed Central PMCID: PMCPMC4596764. doi: 10.1007/s10911-015-9337-0 26188694 10.1007/s10911-015-9337-0 PMC4596764

[33] Llovera M, Pichard C, Bernichtein S, Jeay S, Touraine P, Kelly PA, et al Human prolactin (hPRL) antagonists inhibit hPRL-activated signaling pathways involved in breast cancer cell proliferation. Oncogene. 2000;19(41):4695–705. Epub 2000/10/14. doi: 10.1038/sj.onc.1203846 11032019 10.1038/sj.onc.1203846

[34] Rui H, Lebrun JJ, Kirken RA, Kelly PA, Farrar WL. JAK2 activation and cell proliferation induced by antibody-mediated prolactin receptor dimerization. Endocrinology. 1994;135(4):1299–306. Epub 1994/10/01. doi: 10.1210/endo.135.4.7925093 7925093 10.1210/endo.135.4.7925093

[35] Lee JH, Kim TH, Oh SJ, Yoo JY, Akira S, Ku BJ, et al Signal transducer and activator of transcription-3 (Stat3) plays a critical role in implantation via progesterone receptor in uterus. FASEB journal: official publication of the Federation of American Societies for Experimental Biology. 2013;27(7):2553–63. Epub 2013/03/28. PubMed Central PMCID: PMCPMC3688751. 23531596 10.1096/fj.12-225664 PMC3688751

[36] Suman P, Gupta SK. STAT3 and ERK1/2 cross-talk in leukaemia inhibitory factor mediated trophoblastic JEG-3 cell invasion and expression of mucin 1 and Fos. American journal of reproductive immunology (New York, NY: 1989). 2014;72(1):65–74. Epub 2014/04/11. 10.1111/aji.12248 24716848

[37] Chen CY, Liu SH, Chen CY, Chen PC, Chen CP. Human placenta-derived multipotent mesenchymal stromal cells involved in placental angiogenesis via the PDGF-BB and STAT3 pathways. Biology of reproduction. 2015;93(4):103 Epub 2015/09/12. doi: 10.1095/biolreprod.115.131250 26353894 10.1095/biolreprod.115.131250

[38] Wang W, Guo C, Zhu P, Lu J, Li W, Liu C, et al Phosphorylation of STAT3 mediates the induction of cyclooxygenase-2 by cortisol in the human amnion at parturition. Science signaling. 2015;8(400):ra106 Epub 2015/10/29. doi: 10.1126/scisignal.aac6151 26508788 10.1126/scisignal.aac6151

[39] Yu LJ, Wang B, Parobchak N, Roche N, Rosen T. STAT3 cooperates with the non-canonical NF-kappaB signaling to regulate pro-labor genes in the human placenta. Placenta. 2015;36(5):581–6. Epub 2015/03/17. doi: 10.1016/j.placenta.2015.02.013 25771405 10.1016/j.placenta.2015.02.013

[40] Lim W, Song G. Naringenin-induced migration of embrynoic trophectoderm cells is mediated via PI3K/AKT and ERK1/2 MAPK signaling cascades. Molecular and cellular endocrinology. 2016;428:28–37. Epub 2016/03/21. doi: 10.1016/j.mce.2016.03.018 26994515 10.1016/j.mce.2016.03.018

[41] Sferruzzi-Perri AN, Lopez-Tello J, Fowden AL, Constancia M. Maternal and fetal genomes interplay through phosphoinositol 3-kinase(PI3K)-p110alpha signaling to modify placental resource allocation. Proceedings of the National Academy of Sciences of the United States of America. 2016;113(40):11255–60. Epub 2016/09/14. PubMed Central PMCID: PMCPMC5056071. doi: 10.1073/pnas.1602012113 27621448 10.1073/pnas.1602012113 PMC5056071

[42] Wahane SD, Hellbach N, Prentzell MT, Weise SC, Vezzali R, Kreutz C, et al PI3K-p110-alpha-subtype signalling mediates survival, proliferation and neurogenesis of cortical progenitor cells via activation of mTORC2. Journal of neurochemistry. 2014;130(2):255–67. Epub 2014/03/22. doi: 10.1111/jnc.12718 24645666 10.1111/jnc.12718

[43] Amaral ME, Cunha DA, Anhe GF, Ueno M, Carneiro EM, Velloso LA, et al Participation of prolactin receptors and phosphatidylinositol 3-kinase and MAP kinase pathways in the increase in pancreatic islet mass and sensitivity to glucose during pregnancy. The Journal of endocrinology. 2004;183(3):469–76. Epub 2004/12/14. doi: 10.1677/joe.1.05547 15590973 10.1677/joe.1.05547

[44] Ambros V, Bartel B, Bartel DP, Burge CB, Carrington JC, Chen X, et al A uniform system for microRNA annotation. RNA (New York, NY). 2003;9(3):277–9. Epub 2003/02/20. PubMed Central PMCID: PMCPMC1370393. 10.1261/rna.2183803 PMC1370393 12592000

