# Supplementary material for: Evaluation of circulating miRNAs during late pregnancy in the mare
Source: PLoS One. 2017 Apr 7;12(4):e0175045. doi: 10.1371/journal.pone.0175045 (PMC5384662; doi:10.1371/journal.pone.0175045)
Supplement: S1 Table — Sequences of 178 primers used for qPCR analysis. (DOCX) [file pone.0175045.s004.docx]

| miRNA | Sequence |
| --- | --- |
| eca-let-7a-5p | tgaggtagtaggttgtatagtt |
| eca-let-7c-5p | tgaggtagtaggttgtatggtt |
| eca-let-7d-5p | agaggtagtaggttgcatagtt |
| eca-let-7e-5p | tgaggtaggaggttgtatagtt |
| eca-let-7f-5p | tgaggtagtagattgtatagtt |
| eca-let-7g-5p | tgaggtagtagtttgtacagtt |
| eca-mir-1-3p | tggaatgtaaagaagtatgtat |
| eca-mir-7-5p | tggaagactagtgattttgttgt |
| eca-mir-9a-5p | tctttggttatctagctgtatga |
| eca-mir-10a-5p | taccctgtagatccgaatttgtg |
| eca-mir-18b-5p | taaggtgcatctagtgcagttag |
| eca-mir-19a-3p | tgtgcaaatctatgcaaaactga |
| eca-mir-20a-5p | taaagtgcttatagtgcaggtag |
| eca-mir-20b-5p | caaagtgctcatagtgcaggtag |
| eca-mir-21-5p | tagcttatcagactgatgttga |
| eca-mir-22-3p | aagctgccagttgaagaactgt |
| eca-mir-23a-3p | atcacattgccagggatttcc |
| eca-mir-26a-5p | ttcaagtaatccaggataggct |
| eca-mir-27b-3p | ttcacagtggctaagttctgc |
| eca-mir-30b-5p | tgtaaacatcctacactcagct |
| eca-mir-30d-5p | tgtaaacatccccgactggaag |
| eca-mir-32-5p | tattgcacattactaagttgca |
| eca-mir-33b-5p | gtgcattgctgttgcattgc |
| eca-mir-34-5p | tggcagtgtcttagctggttgt |
| eca-mir-92b-3p | tattgcactcgtcccggcctcc |
| eca-mir-93-5p | caaagtgctgttcgtgcaggtag |
| eca-mir-95-3p | ttcaacgggtctttattgagca |
| eca-mir-99a-5p | aacccgtagatccgatcttgtg |
| eca-mir-99b-5p | cacccgtagaaccgaccttgcg |
| eca-mir-100-5p | aacccgtagatccgaacttgtg |
| eca-mir-103-3p | agcagcattgtacagggctatga |
| eca-mir-106a-5p | caaagtgcttacagtgcaggtag |
| eca-mir-106b-5p | taaagtgctgacagtgcagat |
| eca-mir-124-3p | taaggcacgcggtgaatgcc |
| eca-mir-125a-3p | acaggtgaggttcttgggagcc |
| eca-mir-125a-5p | tccctgagaccctttaacctgtga |
| eca-mir-125b-5p | tccctgagaccctaacttgtga |
| eca-mir-126-3p | tcgtaccgtgagtaataatgcg |
| eca-mir-128-3p | tcacagtgaaccggtctcttt |
| eca-mir-129a-3p | aagcccttaccccaaaaagtat |
| eca-mir-129a-5p | ctttttgcggtctgggcttgc |
| eca-mir-130a-3p | cagtgcaatgttaaaagggcat |
| eca-mir-130b-3p | cagtgcaatgatgaaagggcat |
| eca-mir-132-3p | taacagtctacagccatggtcg |
| eca-mir-133b-3p | tttggtccccttcaaccagcta |
| eca-mir-135a-5p | tatggctttttattcctatgtga |
| eca-mir-136-5p | actccatttgttttgatgatgg |
| eca-mir-137-3p | ttattgcttaagaatacgcgtag |
| eca-mir-138-5p | agctggtgttgtgaatcaggccg |
| eca-mir-140-3p | taccacagggtagaaccacgg |
| eca-mir-140-5p | cagtggttttaccctatggtag |
| eca-mir-146a-5p | tgagaactgaattccatgggtt |
| eca-mir-146b-3p | tgccctagggactcagttctgg |
| eca-mir-146b-5p | tgagaactgaattccataggct |
| eca-mir-147b-3p | gtgtgccgaaatgcttctgcta |
| eca-mir-148a-3p | tcagtgcactacagaactttgt |
| eca-mir-155-5p | ttaatgctaatcgtgataggggt |
| eca-mir-183-5p | tatggcactggtagaattcact |
| eca-mir-188-5p | catcccttgcatggtggaggg |
| eca-mir-190b-3p | tgatatgtttgatattgggtt |
| eca-mir-191-5p | caacggaatcccaaaagcagctg |
| eca-mir-192-5p | ctgacctatgaattgacagcc |
| eca-mir-193a-3p | aactggcctacaaagtcccagt |
| eca-mir-193a-5p | tgggtctttgcgggcgagatga |
| eca-mir-195-5p | tagcagcacagaaatattggc |
| eca-mir-197-3p | ttcaccaccttctccacccagc |
| eca-mir-199b-3p | acagtagtctgcacattggtta |
| eca-mir-200a-3p | taacactgtctggtaacgatgt |
| eca-mir-200b-3p | taatactgcctggtaatgatga |
| eca-mir-200c-3p | taatactgccgggtaatgatgga |
| eca-mir-204b-5p | ttccctttgtcatcctatgcct |
| eca-mir-206-3p | tggaatgtaaggaagtgtgtgg |
| eca-mir-212-3p | taacagtctccagtcacggcc |
| eca-mir-214-3p | acagcaggcacagacaggcagt |
| eca-mir-215-5p | atgacctatgaattgacagac |
| eca-mir-216a-5p | taatctcagctggcaactgtga |
| eca-mir-217-5p | tactgcatcaggaactgattgga |
| eca-mir-218-5p | ttgtgcttgatctaaccatgt |
| eca-mir-219-5p | tgattgtccaaacgcaattct |
| eca-mir-224-5p | caagtcactagtggttccgtt |
| eca-mir-302d-3p | taagtgcttccatgttttagtgt |
| eca-mir-324-5p | cgcatcccctagggcattggtgt |
| eca-mir-328-3p | ctggccctctctgcccttccgt |
| eca-mir-329-3p | aacacacctagttaacctcttt |
| eca-mir-330-5p | tctctgggcctgtgtcttaggc |
| eca-mir-331-3p | gcccctgggcctatcctagaa |
| eca-mir-335-5p | tcaagagcaataacgaaaaatgt |
| eca-mir-342-3p | tctcacacagaaatcgcacccgt |
| eca-mir-361-3p | tcccccaggcgtgattctgattt |
| eca-mir-361-5p | ttatcagaatctccaggggtac |
| eca-mir-367-3p | aattgcactttagcaatggtga |
| eca-mir-369-3p | aataatacatggttgatcttt |
| eca-mir-369-5p | agatcgaccgtgtcatattcgc |
| eca-mir-371-3p | aagtgccgccattttttgagtgt |
| eca-mir-371-5p | actcaaactgtgggggcact |
| eca-mir-374a-5p | ttataatacaacctgataagtg |
| eca-mir-374b-5p | atataatacaacctgctaagtg |
| eca-mir-376a-3p | atcatagaggaaaatccacgt |
| eca-mir-376b-3p | atcatagaggaaaatccatgt |
| eca-mir-377-3p | atcacacaaaggcaacttttgt |
| eca-mir-378-3p | actggacttggagtcagaagg |
| eca-mir-379-5p | tggtagactatggaacgtagg |
| eca-mir-380-3p | tatgtaatatggtccacgtctt |
| eca-mir-382-5p | gaagttgttcgtggtggattcg |
| eca-mir-410-3p | aatataacacagatggcctgt |
| eca-mir-411-5p | tagtagaccgtatagcgtacg |
| eca-mir-412-3p | ttcacctggtccactagccg |
| eca-mir-423-5p | tgaggggcagagagcgagacttt |
| eca-mir-431-5p | tgtcttgcaggccgtcatgcagg |
| eca-mir-432-5p | tcttggagtaggtcattgggtgg |
| eca-mir-433-3p | atcatgatgggctcctcggtgt |
| eca-mir-450b-3p | ttgggaacattttgcatccata |
| eca-mir-454-3p | tagtgcaatattgcttatagggt |
| eca-mir-485-3p | gtcatacacggctctcctctct |
| eca-mir-485-5p | agaggctggccgtgatgaattc |
| eca-mir-486-3p | cggggcagctcagtacaggat |
| eca-mir-486-5p | tcctgtactgagctgccccgag |
| eca-mir-487a-3p | aatcatacagggacatccagtt |
| eca-mir-487b-3p | aatcgtacagggtcatccactt |
| eca-mir-488-3p | ttgaaaggctatttcttggtc |
| eca-mir-489-3p | gtgacatcacatatacggcggc |
| eca-mir-490-5p | ccatggatctccaggtgggt |
| eca-mir-492-5p | aggagctgcgggacaagattctt |
| eca-mir-493b-3p | tgaaggtcttccgtgtgccagg |
| eca-mir-495-3p | aaacaaacatggtgcacttctt |
| eca-mir-496-3p | tgagtattacatggccaatctc |
| eca-mir-497-5p | cagcagcacactgtggtttgt |
| eca-mir-499-3p | aacatcacagcaagtctgtgct |
| eca-mir-499-5p | ttaagacttgcagtgatgttt |
| eca-mir-503-5p | tagcagcgggaacagtactgcag |
| eca-mir-504-5p | agaccctggtctgcactctatc |
| eca-mir-508-3p | tgattgtcaccttttggagtaga |
| eca-mir-514-3p | attgacacctctgtgagtgga |
| eca-mir-532-5p | catgccttgagtgtaggaccgt |
| eca-mir-542-3p | tgtgacagattgataactgaaa |
| eca-mir-542-5p | ctcggggatcatcatgtcacga |
| eca-mir-544b-3p | attctgcatttttaacaagttc |
| eca-mir-551b-3p | gcgacccatacttggtttcag |
| eca-mir-582-3p | taaccggttgaacaactgaacc |
| eca-mir-582-5p | ttacagttgttcaaccagttact |
| eca-mir-590-3p | taattttatgtataagctagt |
| eca-mir-590-5p | gagcttattcataaaagtacag |
| eca-mir-592-5p | ttgtgtcaatatgcgatgatgt |
| eca-mir-598-3p | tacgtcatcgttgtcatcgtca |
| eca-mir-615-5p | gggggtccccggtgctcggatc |
| eca-mir-632-3p | gtgcctgtttcctgtggga |
| eca-mir-655-3p | ataatacatggttaacctcttt |
| eca-mir-664-3p | tattcatttatctcctagcctaca |
| eca-mir-671-5p | aggaagccctggaggggctggag |
| eca-mir-674-3p | aggaggccatagtggcaactgt |
| eca-mir-675-5p | tggcgcggagagggcccacagtg |
| eca-mir-758-3p | tttgtgacctggtccactaacc |
| eca-mir-763-5p | ccagctgggaggaaccagtggc |
| eca-mir-767-3p | tctgctcatactccatggttcct |
| eca-mir-767-5p | tgcaccatggttgtctgagcatg |
| eca-mir-769b-5p | ggaaacctctgggttctgagct |
| eca-mir-770-5p | agcaccacgtgtctgggccatg |
| eca-mir-802-5p | cagtaacaaagattcatccttgt |
| eca-mir-872-5p | aaggttacttgttagttcagg |
| eca-mir-873-5p | gcaggaacttgtgagtctcct |
| eca-mir-876-3p | tggtggtggtttacaaagtaattca |
| eca-mir-885-3p | aggcagcggggtgtagtggata |
| eca-mir-885-5p | tccattacactaccctgcctct |
| eca-mir-889-3p | ttaatatcggacaaccattgt |
| eca-mir-1185-5p | agaggataccctttgtatgtt |
| eca-mir-1193-3p | taggtcacccgtttgactatc |
| eca-mir-1282-5p | agtggttggtttgtatgagatggtt |
| eca-mir-1289-3p | tggagtccaggaatctgcatttt |
| eca-mir-1296-5p | ttagggccctggctccatctcc |
| eca-mir-1298-5p | ttcattcggctgtccagatgta |
| eca-mir-1302-3p | ttgggacatacttatactaaa |
| eca-mir-1302c-3p | ttgcgacatacttatactaaa |
| eca-mir-1302e-3p | ttgggatatacttatactaaa |
| eca-mir-1461-5p | atctctacgggtaagtgtgtga |
| eca-mir-1839-5p | aaggtagatagaacaggtcttg |
| eca-mir-1842-5p | tggctctgtgaggtcggctca |
| eca-mir-1898-5p | aggtcaaggttcacaggggatc |
| eca-mir-1902-3p | agaggtgcagtaggcatgactt |
